# Supplementary material for: Effect of Local Topography on Cell Division of Staphylococcus spp
Source: Nanomaterials (Basel). 2022 Feb 18;12(4):683. doi: 10.3390/nano12040683 (PMC8877970; doi:10.3390/nano12040683)
Supplement: Supplementary file 1 [file nanomaterials-12-00683-s001.zip › nanomaterials-1540097-supplementary.pdf]

SUPPLEMENTARY MATERIALS

Effect of Local Topography on Cell Division of *Staphylococcus* spp.

Ioritz Sorzabal-Bellido [1,†], Luca Barbieri [1,2], Alison J. Beckett [3], Ian A. Prior [3], Arturo Susarrey-Arce [4], Roald M. Tiggelaar [5], Joanne Fothergill [2], Rasmita Raval [1,*] and Yuri A. Diaz Fernandez [1,*]

[1] Surface Science Research Centre and Open Innovation Hub for Antimicrobial Surfaces, Department of Chemistry, University of Liverpool, Liverpool L69 7ZD, UK; isorzabal@unav.es (I.S.-B.); luca.barbieri@liverpool.ac.uk (L.B.);
[2] Institute of Infection and Global Health, University of Liverpool, Liverpool L69 3BX, UK; j.fothergill@liverpool.ac.uk
[3] Biomedical Electron Microscopy Unit, University of Liverpool, Liverpool L69 3BX, UK; alib@liverpool.ac.uk (A.J.B.); iprior@liverpool.ac.uk (I.A.P.)
[4] Mesoscale Chemical Systems, MESA+ Institute, University of Twente, 7522 NB Enschede, The Netherlands; a.susarreyarce@utwente.nl
[5] NanoLab cleanroom, MESA+ Institute, University of Twente, 7522 NB Enschede, The Netherlands; r.m.tiggelaar@utwente.nl
* Correspondence: raval@liverpool.ac.uk (R.R.); yuridiaz@liverpool.ac.uk (Y.A.D.F)
† Current address: IDISNA, Ciberonc and Solid Tumors and Biomarkers Program, Center for Applied Medical Research, University of Navarra, Navarra, Spain.

S1 Additional SEM Images of *Staphylococcus* spp. on Flat Si and SiNWs Surfaces

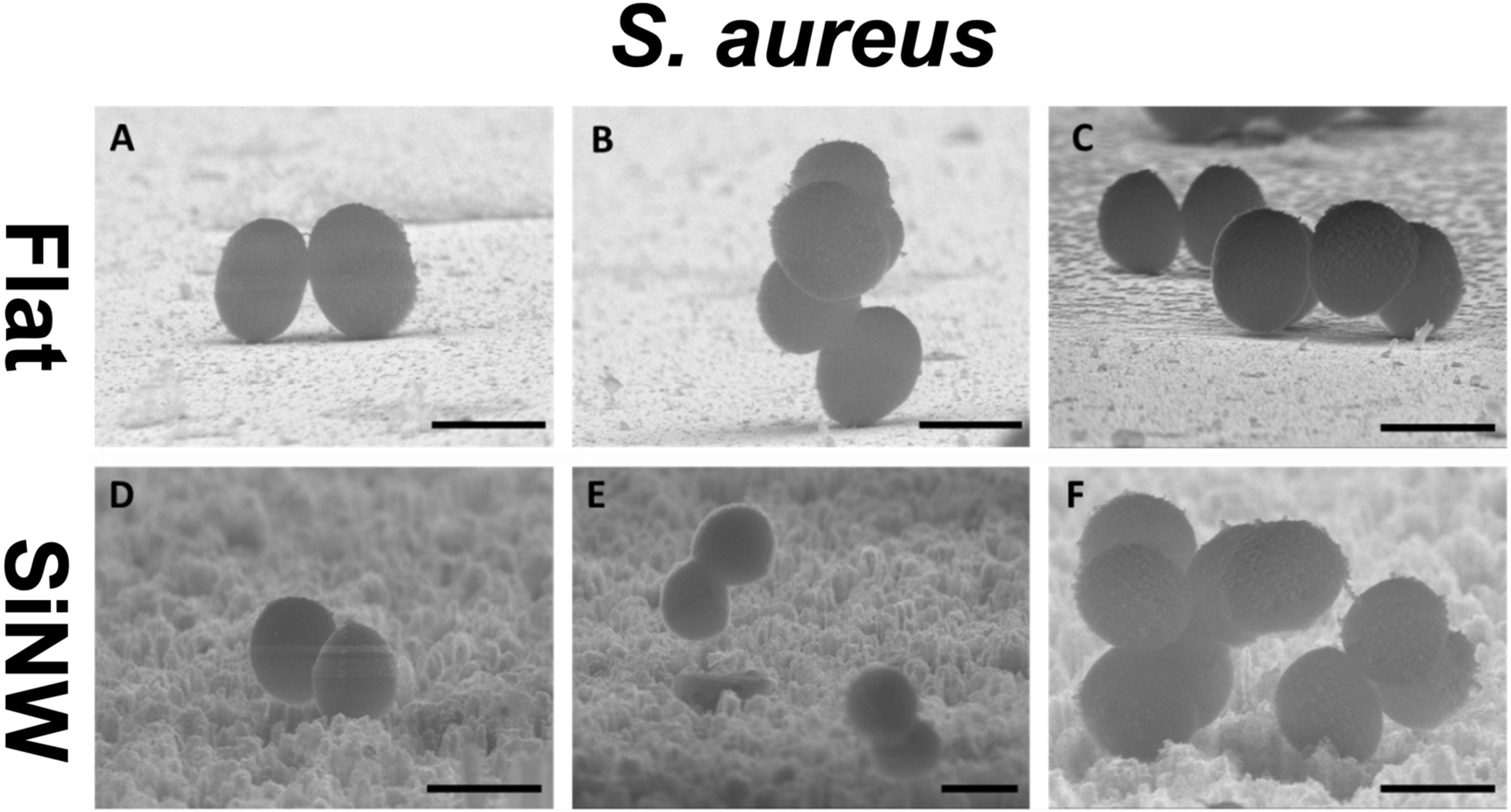

Figure S1. Representative SEM images of *S. aureus* on flat Si (A, B, C) and SiNWs (D, E, F) surfaces. Scale bars are 1 μm.

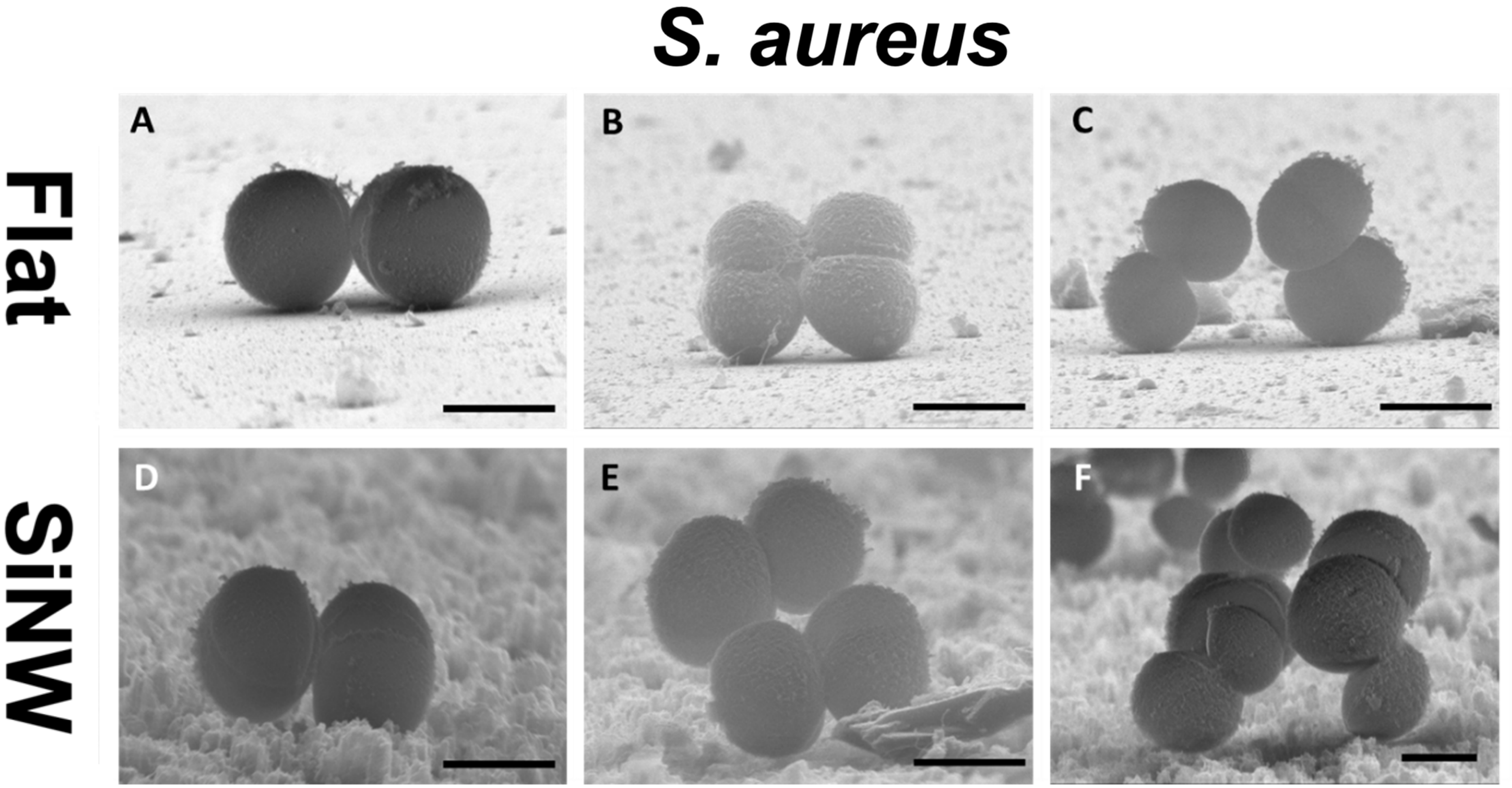

Figure S2. Representative SEM images of *S. aureus* on flat Si (A, B, C) and SiNWs (D, E, F) surfaces. Scale bars are 1 μm.

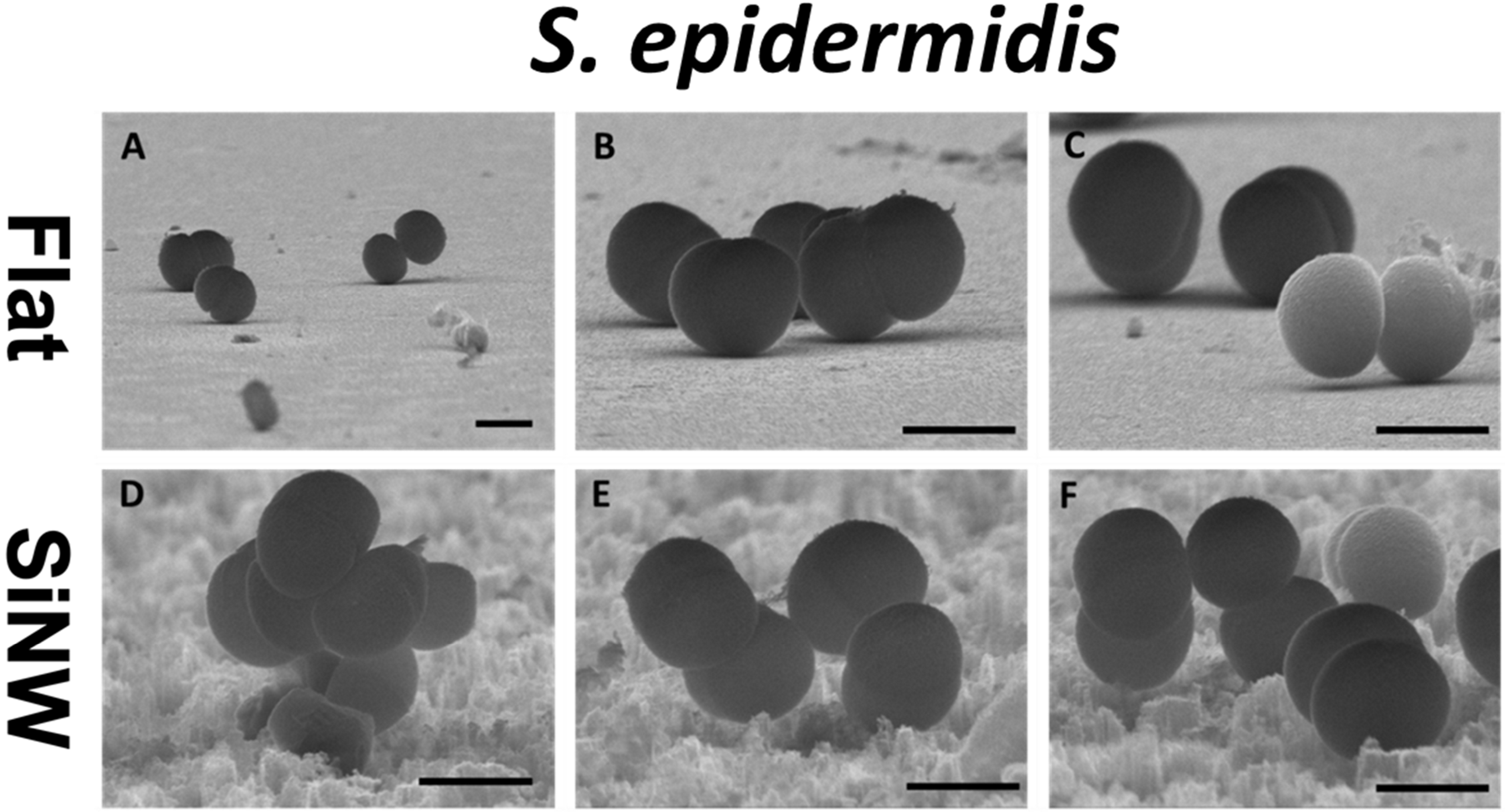

Figure S3. Representative SEM images of *S.epidermidis* on flat Si (A, B, C) and SiNWs (D, E, F) surfaces. Scale bars are 1 μm.

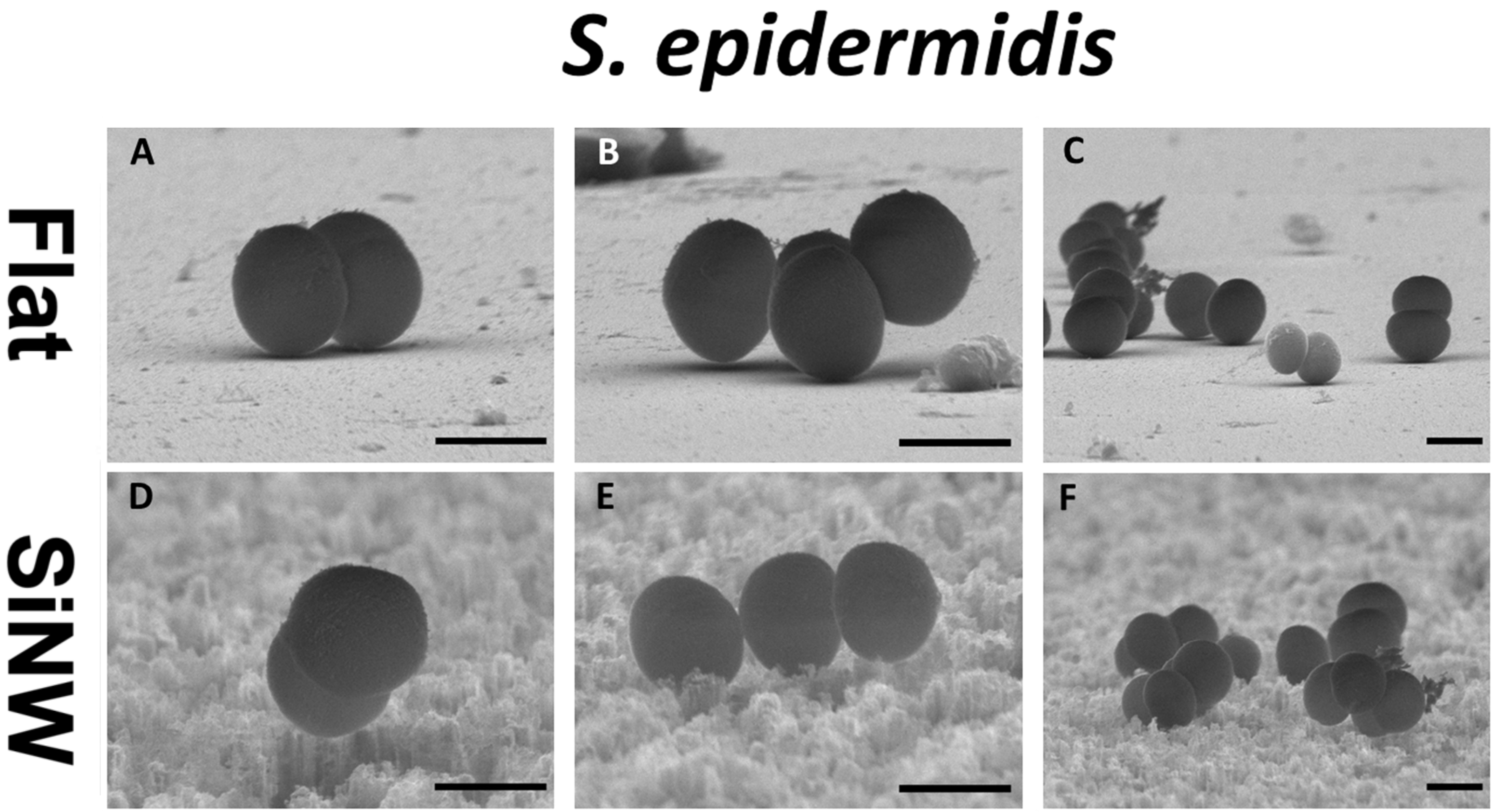

Figure S4. Representative SEM images of *S.epidermidis* on flat Si (A, B, C) and SiNWs (D, E, F) surfaces. Scale bars are 1 μm

S2 Topographic Analysis of SiNWs Surfaces

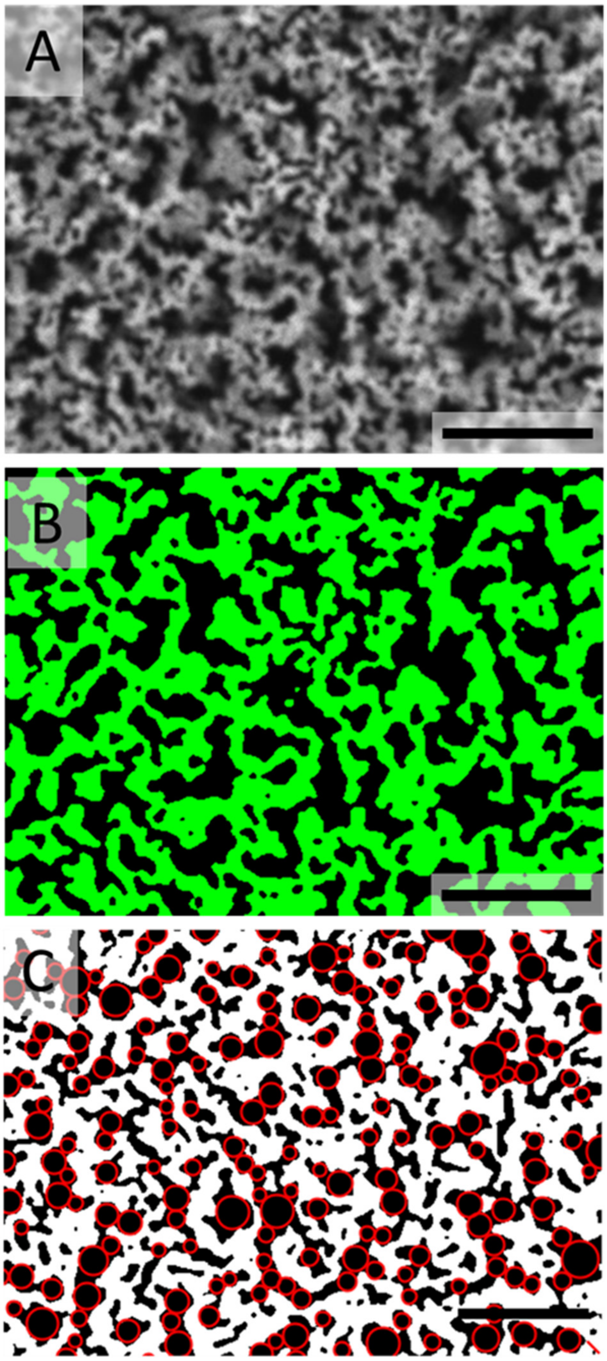

Figure S5 Representative sequence of imaging processing of SEM data for SiNWs surfaces: A) Original SEM image; B) Binary mask generated by ImageJ's *Default* threshold; C) pore size analysis using largest inscribed circle fitting, (reference to https://github.com/BIOP/ijp-max-inscribed-circles; accessed on 15 January 2022). Scale bar is 2 μm.

Table S1 Statistical descriptors for the weighted distributions of pore size r_{pore}, fraction of free cell area f_{growth}, average vertical offset $\Delta z_{average}$, and critical angle θ_{max} for the SiNWs surfaces. On this table: "sem" stands for "standard error of the mean" and QD for "quartile deviation". All the distributions are weighted by the area of the pore (i.e. weighting factor = $\pi(r_{pore})^2$)

	**Mean**	**sem**	**Median**	**QD**	**Q25**	**Q75**
r_{pore}	153 nm	0.4 nm	142 nm	27 nm	122 nm	176 nm
f_{growth}	0.937	0.003	0.950	0.02	0.925	0.961
$\Delta z_{average}$	755 nm	0.3 nm	747 nm	20 nm	731 nm	772 nm
Δz_{min}	306nm	0.8nm	284nm	54nm	244nm	352nm

S3 Geometric Formulations

In this section we will discuss some considerations that allowed us to calculate the different topographical parameters determining cell growth and micro-colony architecture as a function of the local topography. On the basis of purely geometrical considerations, we calculated the topographically hindered area of a quasi-spherical cell of radius R_{cell}, interacting with a topographic cavity of size r_{pore} as follows:

$$a_{blocked} = 2\pi \cdot R_{cell} \cdot h \tag{S1}$$

where h is defined in Figure S6, and the following geometrical relationships apply:

$$R_{cell} = h + \delta \tag{S2}$$

$$(R_{cell})^2 = \delta^2 + (r_{pore})^2 \tag{S3}$$

$$h = R_{cell} - \sqrt{(R_{cell})^2 - (r_{pore})^2} \tag{S4}$$

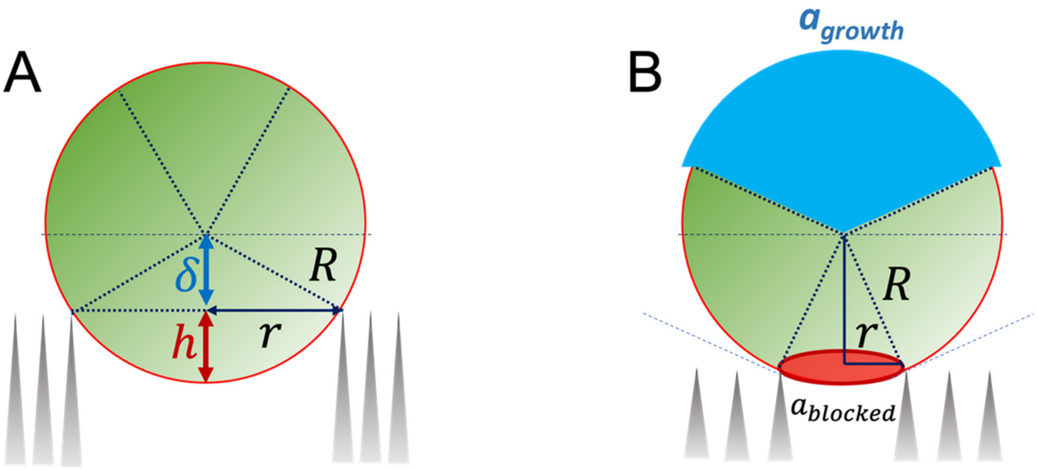

Figure S6. Geometric considerations used for the calculation of f_{growth}.

Subsequently, we defined the fraction of blocked area in the lower cell hemisphere:

$$f_{blocked} = \frac{a_{blocked}}{2\pi \cdot (R_{cell})^2} \tag{S5}$$

And using Equations (S2)-(S4)we obtained:

$$f_{growth} = \sqrt{1 - \frac{(r_{pore})^2}{(R_{cell})^2}} \tag{S6}$$

From here, we obtained the fraction of free area of the cell f_{growth}, assuming $f_{blocked} + f_{growth} = 1$, and leading to:

$$f_{growth} = \sqrt{1 - \frac{(r_{pore})^2}{(R_{cell})^2}} \qquad \text{(S7)}$$

This result implies that on a flat surface, where there are not topographic cavities (i.e. $r_{pore} = 0$), the fraction of topographically blocked surface is $f_{blocked} \approx 0$ and therefore $f_{growth} \approx 1$ and no growth inhibition caused by a chemically inert surface can be predicted. Conversely, in the presence of a topographic feature of finite size, like those of SiNWs surfaces, $f_{growth} < 1$ and the surface may display some bacteriostatic effect due to hindrance of growth when the 1st generation division septum lays within the blocked fraction of the cell surface. The extreme case $f_{growth} \approx 0$ may occur when the size of the topographic feature is similar to the size of the cell (i.e. $r_{pore} \approx R_{cell}$), however for our vertically aligned SiNWs arrays, this event was found to be unlikely due to the specific pore size distribution of the topography, showing pore sizes smaller than the characteristic size of *Staphylococcus* cells.

The hindered area on the cell surface reduced also the angular range where cell growth and colony development can occur. The maximum azimuthal angle θ_{max} at which a 1st generation daughter cell can growth was determined by the expression:

$$\theta_{max} = arcos\left(\frac{r_{pore}}{R_{cell}}\right) \qquad \text{(S8)}$$

This expression led to the trivial solution $\theta_{max} \approx \pi/2$ for a flat surface (i.e. $r_{pore}=0$), suggesting that the 1st generation cells can lay perpendicular to the surface normal and therefore completely horizontal growth is allowed, in agreement with experimental observations of *Staphylococcus* cells on flat Si (main text Figure2, and Figure S1 A,B and Figure S2 B,C). On the other hand, in the presence of a topographic feature of finite size, like on SiNWs surfaces, $\theta_{max} < \pi/2$ due to the effect of the topographic

hindrance, forcing the 1st generation daughter cells to grow further away from the surface plane (Figure S1 E,F and Figure S2 D,E). Another way of representing this effect is by calculating the displacement in the vertical axis, perpendicular to the surface plane, induced by the angular constrain. For any azimuthal angle θ, the displacement in the vertical axis Δz can be calculated according to Figure S7 as:

$$\Delta z = 2 \cdot R_{cell} \cdot \cos\theta \qquad \text{(S9)}$$

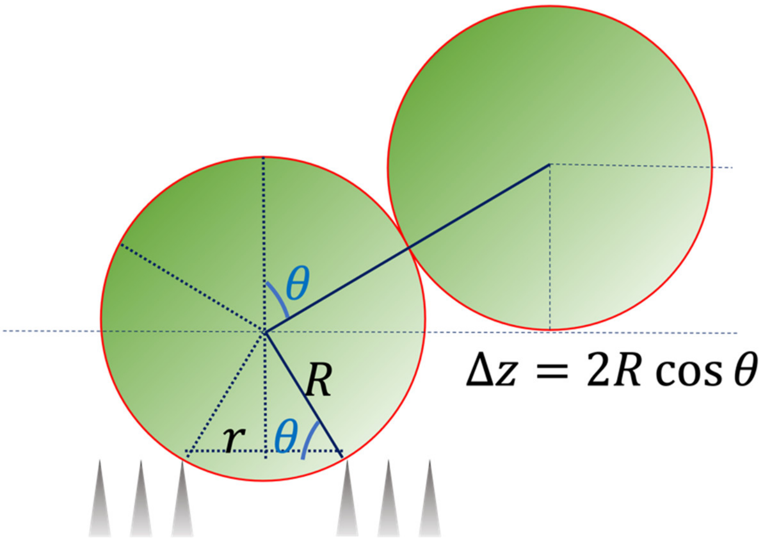

Figure S7 Geometrical consideration used for the calculation of the vertical displacement Δz of a daughter cell as a function of the azimuthal angle θ.

In the case of the flat surface, θ took any value between 0 and $\pi/2$, and therefore Δz varied from $2R_{cell}$ to zero. On SiNWs, as discussed above, the angular range is reduced, and therefore Δz is always greater than zero, reaching a minimum value for $\theta=\theta_{max}$ at $\Delta z_{min}=2r_{pore}$. Therefore, on a porous surface such as our SiNWs, the 1st generation daughter cells will always be pushed away from the surface plane, having direct implications on the topological model discussed in the main text. Furthermore, we were able to calculate the average values of Δz by integration over the azimuth angle θ:

$$\Delta z_{average} = 2 \cdot R_{cell} \cdot \frac{\int_0^{\theta_{max}} \cos\theta \, d\theta}{\int_0^{\theta_{max}} d\theta} \tag{S10}$$

$$\Delta z_{average} = \frac{2 \cdot R_{cell} \cdot \sin(\theta_{max})}{\theta_{max}} \tag{S11}$$

Equations (S7), (S8) and (S11) were used to determine the probability density functions of the different topographical parameters as a function of the pore size distribution of SiNWs surfaces, weighting by the area of the pore to account for the fact that although bigger pores were less abundant, they displayed intrinsically a higher cross-section of interaction with *Staphylococcal* cells. These calculated probability density functions, including those of the surface pore size, are presented in the main text Figures 3D, 5E, and 6B, and some key statistical descriptors are reported in Supplementary Materials Table S1.

S4 Morphological characterisation of *Staphylococcus* spp. micro-colonies on Flat Si and SiNWs surfaces

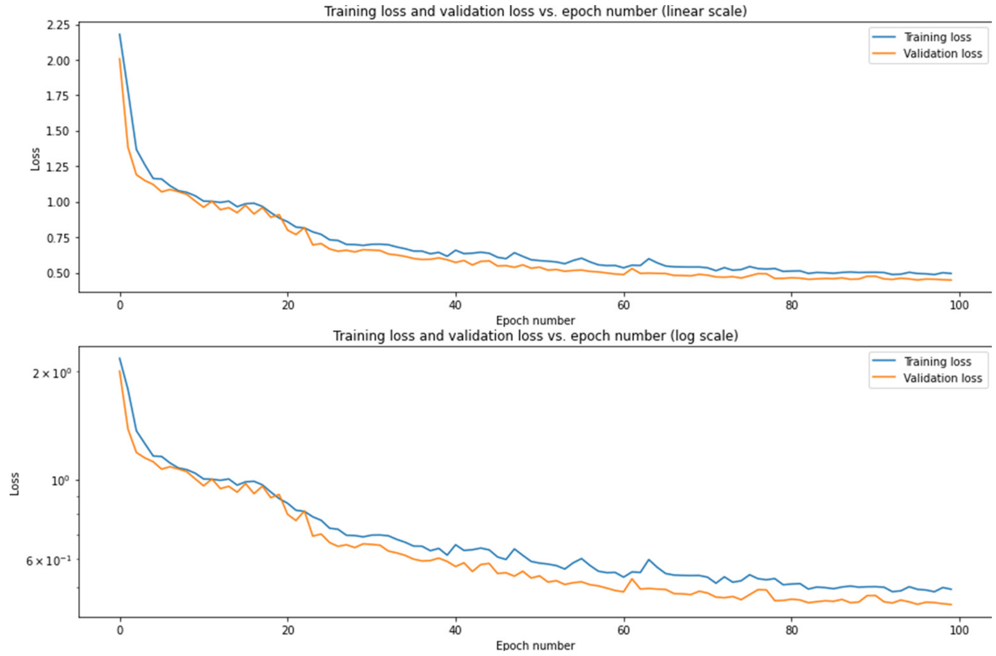

Figure S8. StarDist 2D model optimisation process measured as training and validation loss per number of training epochs.

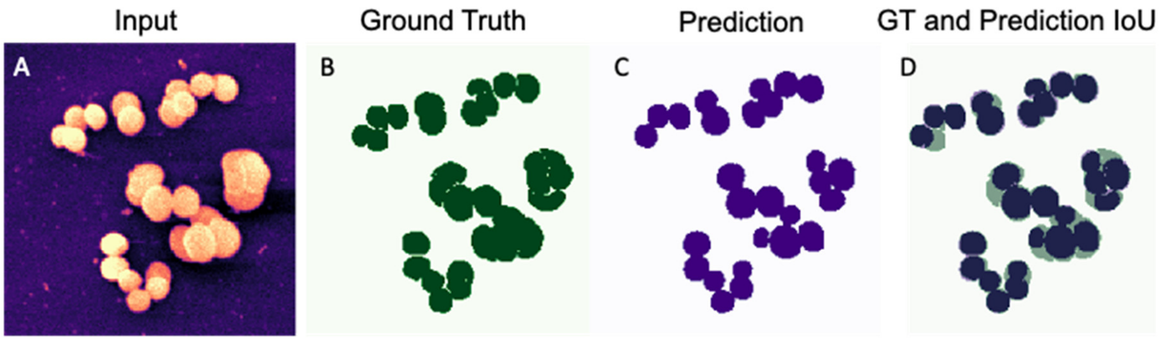

E

IoU	Precision	Recall	Accuracy	F1
0.84±0.04	0.99±0.02	0.92±0.04	0.91±0.05	0.95±0.03

Figure S9. Quality control report of *S.aureus* StarDist2D segmentation model: A) Input image, B) ground truth target, C) predicted segmentation and D) ground truth and prediction intersection over union (IoU). E) Quality control metrics for S. aureus StarDist2D segmentation model.

Table S2 Statistical descriptors for the distribution of morphological parameters of *S. aureus* colonies on Flat Si and SiNWs surfaces. On this table: "sem" stands for "standard error of the mean" and QD for "quartile deviation".

		Mean	sem	Median	QD	Q25	Q75
**Flat**	N_{cells} $per\ colony$	12.0	0.1	8.0	6.0	4.0	16.0
	$Circularity$	0.63	0.005	0.67	0.14	0.51	0.79
	$Feret's\ diameter$	3.2 μm	0.05 μm	2.7 μm	0.85 μm	2.1 μm	3.8 μm

		Mean	sem	Median	QD	Q25	Q75
**SiNW**	N_{cells} $per\ colony$	6.8	0.1	5.0	3.0	3.0	9.0
	$Circularity$	0.68	0.005	0.74	0.12	0.56	0.81
	$Feret's\ diameter$	2.9μm	0.03 μm	2.5μm	0.65 μm	2.1μm	3.4μm

S5 Imaging processing details

Table S3. Advanced parameter configuration used for *S. aureus* SEM segmentation model training.

**Parameter**	**Value**
number_of_epochs	100
patch_size	176x176
batch_size	2
number_of_steps	20
percentage_validation	10
n_rays	32
grid_parameter	2
initial_learning_rate	0.0003

Table S4. Key Python packages used for *S. aureus* SEM segmentation model training.

**Python Package**	**Version**
Tensorflow	v0.1.12
Keras	v2.3.1
CSBDeep	v0.6.1
Numpy	v1.19.5
Cuda	v11.0.221

S6 Average Number of Anchoring Points as a Function of the Number of Cells.

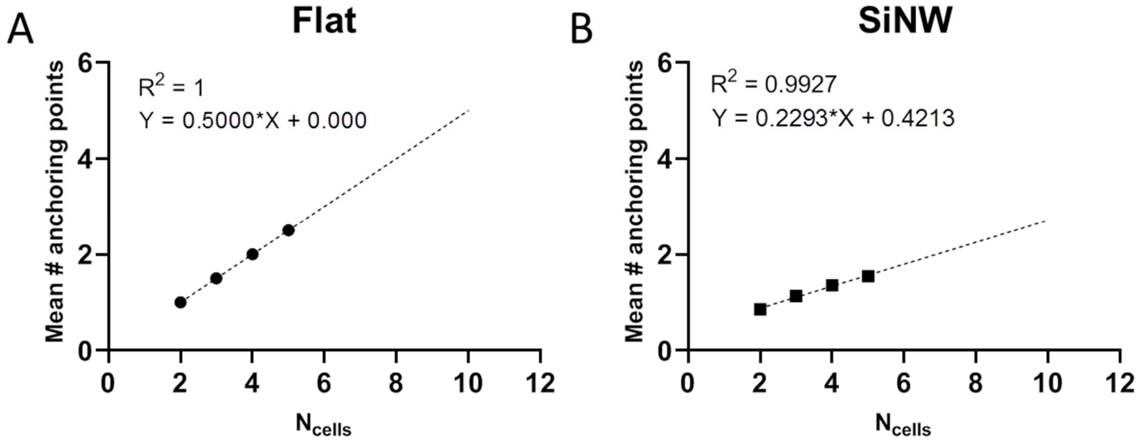

Figure S10. Numerical fittings for the average number of anchoring points, calculated from the sub-family of allowed adjacency matrixes, as a function of the number of cells for: A) Flat Si; B) SNWs surface. Best fitting equation and regression coefficient are reported within the text inset.
